# Integrative analysis of *Lunatic Fringe* variants associated with spondylocostal dysostosis type‐III

**DOI:** 10.1096/fj.202302651RR

**Published:** 2024-06-25

**Authors:** Parker Wengryn, Felicity Fenrich, Karina da Costa Silveira, Connor Oborn, Shuji Mizumoto, Alexander Beke, Carrie‐Lynn Soltys, Shuhei Yamada, Peter Kannu

**Affiliations:** ^1^ Department of Medical Genetics University of Alberta Edmonton Alberta Canada; ^2^ Department of Medicine University of Alberta Edmonton Alberta Canada; ^3^ Department of Molecular and Cellular Biology University of Guelf Guelf Ontario Canada; ^4^ Department of Pathobiochemistry Meijo University Nagoya Aichi Japan

**Keywords:** Dysostoses, embryology, LFNG, rare diseases, Somites, spine

## Abstract

*Lunatic Fringe* (*LFNG*) is required for spinal development. Biallelic pathogenic variants cause spondylocostal dysostosis type‐III (SCD3), a rare disease generally characterized by malformed, asymmetrical, and attenuated development of the vertebral column and ribs. However, a variety of SCD3 cases reported have presented with additional features such as auditory alterations and digit abnormalities. There has yet to be a single, comprehensive, functional evaluation of causative *LFNG* variants and such analyses could unveil molecular mechanisms for phenotypic variability in SCD3. Therefore, nine *LFNG* missense variants associated with SCD3, c.564C>A, c.583T>C, c.842C>A, c.467T>G, c.856C>T, c.601G>A, c.446C>T, c.521G>A, and c.766G>A, were assessed in vitro for subcellular localization and protein processing. Glycosyltransferase activity was quantified for the first time in the c.583T>C, c.842C>A, and c.446C>T variants. Primarily, our results are the first to satisfy American College of Medical Genetics and Genomics PS3 criteria (functional evidence via well‐established assay) for the pathogenicity of c.583T>C, c.842C>A, and c.446C>T, and replicate this evidence for the remaining six variants. Secondly, this work indicates that all variants that prevent Golgi localization also lead to impaired protein processing. It appears that the FRINGE domain is responsible for this phenomenon. Thirdly, our data suggests that variant proximity to the catalytic residue may influence whether LFNG is improperly trafficked and/or enzymatically dysfunctional. Finally, the phenotype of the axial skeleton, but not elsewhere, may be modulated in a variant‐specific fashion. More reports are needed to continue testing this hypothesis. We anticipate our data will be used as a basis for discussion of genotype–phenotype correlations in SCD3.

## Graphical abstract

Molecular categorization of *Lunatic Fringe (LFNG)* variants which cause spondylocostal dysostosis type‐III. Each category is characterized by the variants’ effect on subcellular localization and enzymatic functionality. We hypothesize that the summative effect of variants on these two characteristics may contribute to a genotype–phenotype correlation where the decreased ability of LFNG to glycosylate NOTCH1 increases the number and severity of vertebral abnormalities. More variants and probands will need to be characterized to test this hypothesis more rigorously. Created with BioRender.com.

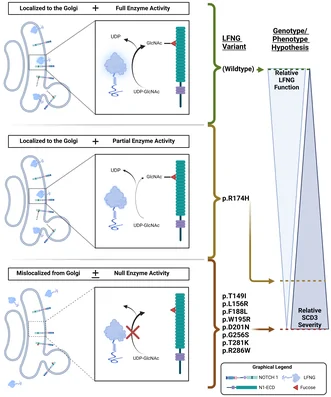

**Abbreviations**
ACMG: American College of Medical Genetics and Genomics
DLL3: delta‐like ligand 3
ER: endoplasmic reticulum
GERP: genomic evolutionary rate profiling
GlcNAc: *N*‐acetylglucosamine
GLT8D1: glcosyltransferase 8 domain‐containing protein 1
GM130: golgi matrix protein 130
GnomAD 3.1.2: Genome Aggregation Database (version 3.1.2)
HES7: hairy enhancer of split 7
LFNG: lunatic fringe
LOVD ID: Leiden Open Variation Database Identifier
MESP2: mesoderm posterior protein 2
MFNG: Manic Fringe
pLDDT: predicted local distance difference test
PPT: pre‐pro‐trypsin
PPT‐PC: pre‐pro‐trypsin leader sequence in plasmid construct
PRRS1: mouse anionic trypsin 2
REVEL: Rare Exome Variant Ensembl Learner
RFNG: radical fringe
RMSD: root‐mean‐square of atomic deviation
SCD3: spondylocostal dysostosis type‐III
SDV: segmentation defect of the vertebrae
SNPdb‐ID: Single Nucleotide Polymorphism Database Identifier
SPC6: subtilisin‐like proteinase 6
TBX6: T‐box transcription factor 6
WT: wild‐type

## 1 INTRODUCTION

The structure of vertebrae and ribs directly results from the symmetrical formation of their precursor structures, somites, in a process called somitogenesis. Somitogenesis occurs early in human embryogenesis and is tightly regulated by the segmentation clock.^1^, ^2^, ^3^, ^4^, ^5^, ^6^, ^7^ A clock cycle lasts 6 h and results in the formation of a new pair of somites which bud bilaterally from the paraxial mesoderm.^5^, ^6^, ^8^ Somitogenesis progresses in a rostrocaudal fashion until all somitic pairs are formed.

The NOTCH signaling pathway is integral to the control of the clock mechanism and thus regulates somitogenesis.^9^, ^10^ *Lunatic Fringe* (*LFNG*), a Golgi‐resident β‐1,3‐*N*‐acetylglucosaminyltransferase, modifies the NOTCH receptor extracellular domain by transferring *N*‐acetylglucosamine (GlcNAc) to specific fucose residues.^1^, ^11^, ^12^, ^13^, ^14^ The glycosylation process modifies how NOTCH receptors interact with NOTCH ligands, essentially recalibrating the signaling mechanism. Resetting the clock mechanism is pivotal for establishing both the anterior and posterior intra‐somitic halves, which are crucial for the development of a symmetrical spondylocostal axis.^15^, ^16^, ^17^

Finely tuned regulation of *LFNG* expression and LFNG processing is required to ensure equal somitic partitioning.^17^, ^18^ LFNG is initially synthesized as a 43 kDa pre‐pro‐protein with two key regions: an N‐terminal, type‐II transmembrane domain (amino acids 1–86), and an enzymatically active FRINGE domain (amino acids 108–358).^13^, ^14^, ^19^ Following translation, LFNG is relocated to the Golgi apparatus, where its transmembrane region serves as an anchor for its FRINGE domain, which faces the Golgi lumen. It is at this location that LFNG exerts its influence on NOTCH receptors. Subtilisin‐like proteinase 6 (SPC6) cleaves the hydrophobic tether at amino acid 86, liberating the processed LFNG (34 kDa) into the extracellular environment within a brief period.^17^, ^18^ This exocytosis process finely tunes segmentation clock timing, facilitating the symmetrical formation of somites.

Biallelic pathogenic variants in *LFNG* cause spondylocostal dysostosis type‐III (SCD3) (OMIM: 609813).^20^, ^21^, ^22^, ^23^, ^24^, ^25^ A subtype of the larger SCD disease family,^9^, ^26^ SCD3 is characterized by short stature and segmentation defects of the vertebrae (SDV) including hemivertebrae, block vertebrae, missing vertebrae, scoliosis, and anomalous rib anatomy.^27^ These changes result in scoliosis and affect the development of the thorax, compromising lung development and potentially leading to restrictive lung disease. Genotyping is often required to distinguish between SCD subtypes due to their phenotypic similarities. Within SCD3, variable presentation includes the prevalence and severity of scoliosis, vertebral number, and the location of the spinal segmentation defects.^22^, ^23^, ^24^ Less frequent physical findings include arachnodactyly, camptodactyly, and auditory impairment.^20^, ^25^, ^28^ Perinatal mortality is uncommon with most affected individuals surviving to adulthood. Previous work has suggested that different *LFNG* variants may modulate phenotypic presentation.^24^ There, it was hypothesized that a hypomorphic *LFNG* genotype resulted in relatively increased activity that would have damaged vertebral column formation less than a null genotype. However, with only 13 cases published in the scientific literature at the time of writing,^20^, ^21^, ^22^, ^23^, ^24^, ^25^, ^28^ it is difficult to draw firm conclusions from the limited data.

The genotypic and functional data of nine *LFNG* missense variants associated with SCD3 published prior to 2023 are listed in Tables 1 and 2. Analysis of potential genotype–phenotype correlations centers on four aspects of the LFNG enzyme: (1) glycosyltransferase activity, (2) protein localization, (3) protein processing, (4) NOTCH signaling activation.^20^, ^22^, ^23^, ^24^ Previous research has primarily utilized the first three approaches to investigate *LFNG* variant pathogenicity as these can provide a specific mechanism for the fourth. However, not all approaches have been used consistently across the published *LFNG* variants. In this study, we aim to functionally characterize nine *LFNG* missense variants associated with SCD3 published with radiographic findings (Tables 1 and 2) in terms of glycosyltransferase activity, subcellular localization, and protein processing. The goal is to provide in vitro evidence that gauges the relative function of each *LFNG* variant, filling experimental gaps from previously published literature to provide general correlations with available SCD3 phenotypic data. We hope that this will form the basis of future work aimed at correlating updated, thorough, and uniform clinical data with the molecular findings collected here, initiating discussion surrounding the genotype–phenotype relationship.

**TABLE 1:** LFNG variants of interest and genetic data.

Publication	Variant	DNA change (GRCh38)	ClinVar ID	SNPdb ID	LOVD DB ID	gnomAD 3.1.2	Splice AI
Sparrow et al. 2006	c.564C>A	NC_000007.14: g.2525301C>A	6999	rs104894024	LFNG_000030	0.00	0.01
Lefebvre et al. 2018	c.583T>C	NC_000007.14: g.2525415T>C	N/A	N/A	N/A	0.00	0.00
Lefebvre et al. 2018	c.842C>A	NC_000007.14: g.2526264C>A	N/A	N/A	N/A	0.00	0.01
Takeda et al. 2018	c.467T>G	NC_000007.14: g.2524729T>G	N/A	N/A	N/A	0.00	0.00
Takeda et al. 2018	c.856C>T	NC_000007.14: g.2526278C>T	N/A	rs752671299	N/A	0.00	0.00
Otomo et al. 2019	c.601G>A	NC_000007.14: 2525432:G>A	619139	rs1211456697	LFNG_000032	0.00	0.00
Schuhmann et al. 2021	c.446C>T	NC_000007.14: g.2524708C>T	N/A	rs867769213	N/A	0.00	0.00
Wengryn et al. 2023	c.521G>A	NC_000007.14: g.2525258G>A	1062453	N/A	LFNG_000034	6.57 × 10^−6^	0.01
Wengryn et al. 2023	c.766G>A	NC_000007.14: g.2525715G>A	1003507	rs1437427476	LFNG_000035	6.57 × 10^−6^	0.07

Abbreviations: gnomAD 3.1.2, Genome Aggregation Database (version 3.1.2); LOVD ID, Leiden Open Variation Database Identifier; SNPdb‐ID, Single Nucleotide Polymorphism Database Identifier.

**TABLE 2:** LFNG variants of interest with structural and functional consequences in silico and in vitro.

Publication	Substitution	REVEL	GERP	RMSD	Predicted Structural Change?	Predicted Distance (Å) to p.D290	Predicted Distance (Å) to p.D201	NOTCH Signalling Assay Activity	Enzyme Activity	Subcellular Localization	PPT‐PC Secretion	Nterminal Cleaved
Sparrow et al. 2006	p.F188L	0.51	4.93	N/A	No^b^	16.1	17.8	Null	Null^b^	Mislocalized^a^	No^b^	No
Lefebvre et al. 2018	p.W195R	0.90	5.32	0.392	Yes	16.8	19.0	N/A	Null	Mislocalized	No	No
Lefebvre et al. 2018	p.T281K	0.62	4.70	0.476	Yes	11.8	24.5	N/A	Null	Mislocalized	No	No
Takeda et al. 2018	p.L156R	0.74	4.83	0.521	Yes	26.1	16.3	N/A	Null^b^	Mislocalized	No^b^	No
Takeda et al. 2018	p.R286W	0.73	4.70	N/A	No	11.7	24.2	N/A	Null^b^	Mislocalized + Golgi	Yes^b^	Yes
Otomo et al. 2019	p.D201N	0.84	5.32	0.318	No	15.3	0.0	N/A	Null^b^	Mislocalized	No^b^	No
Schuhmann et al. 2021	p.T149I	0.68	3.95	0.542	Yes	17.3	10	N/A	Partial	Mislocalized	No	No
Wengryn et al. 2023	p.R174H	0.52^b^	5.18	N/A	No^b^	10.1	17.4	N/A	Partial^b^	Golgi^a^	Yes^b^	Yes^a^
Wengryn et al. 2023	p.G256S	0.89^b^	5.31	0.285	No	8.7	8.6	N/A	Null^b^	Golgi^a^	Yes^b^	Yes^a^

*Note*: All structural predications were generated with Alpha‐Fold. Residue distance predictions were measured from α‐carbon to α‐carbon with the WT model.

Abbreviations: GERP, genomic evolutionary rate profiling; PPT‐PC, pre‐pro‐trypsin leader sequence in plasmid construct; REVEL, Rare exome variant ensemble learner; RMSD, root‐mean‐square of atomic deviation.

^a^ Supporting previous evidence.

^b^ Documented elsewhere.

## 2 MATERIALS AND METHODS

### 2.1 Cloning and subcloning

The creation of the *3XFLAG‐LFNG* plasmid construct is described elsewhere.^23^, ^24^ The creation of the *LFNG‐HA* plasmid construct is also described elsewhere.^24^ Site‐directed mutagenesis (QuikChange XL Site‐Directed Mutagenesis Kit, Agilent, Santa Clara, CA, USA, Cat: 200516–5) was undertaken with both constructs to obtain the desired point variants (see Table S1) following the manufacturer's protocol. We then transformed Mach1 T1 phage‐resistant chemically competent *E. coli* (Thermo Fisher Scientific, Whitby, ON, CAN, Cat: C86203) and extracted clonal plasmid DNA by miniprep (Wizard Plus SV Miniprep DNA Purification System, Promega, Madison, WI, USA, Cat: A1460). The plasmids were screened for point variant induction and random mutagenesis with Sanger sequencing (University of Alberta Molecular Biology Core).

### 2.2 Cell culture

The cell culture protocols for NIH‐3T3 (ATCC, Manasses, VI, USA, Cat: CRL‐1658, RRID:CVCL_0594) are described elsewhere.^24^ COS‐7 cells (JCRB Cell Bank, Osaka, JPN, Cat: JCRB9127, RRID:CVCL_0224) were cultured in high glucose DMEM (FujiFilm Waco, Osaka, JPN, Cat: 044–29765) and 10% FBS (Hyclone, Logan, UT, USA, Cat: SH30396.03). All cell lines were tested for mycoplasma contamination via PCR.

### 2.3 In silico genetic analysis

Rare Exome Variant Ensembl Learner (REVEL), Genomic Evolutionary Rate Profiling (GERP), and SpliceAI scores were obtained for each of the nine genetic variants through the web tool Franklin (Genoox, Palo Alto, CA, USA, https://franklin.genoox.com/clinical‐db/home, RRID:SCR_024670).

### 2.4 In silico protein analysis

The protocol for proteomic analysis is described in our prior publication.^24^ Briefly, wild‐type and variant FASTA files were fed into ColabFold v1.5.2 without any relaxation steps and the top‐ranked structure for each variant was chosen for visual analysis based on predicted local distance difference test (pLDDT) score. Visual analysis was performed in the PyMOL Molecular Graphics System, Version 2.5 (Schrödinger, New York, NY, USA, RRID:SCR_000305).

### 2.5 Ortholog/paralog comparisons

Accession codes for LFNG, (Manic Fringe) MFNG, and Radical Fringe (RFNG) were retrieved from UniprotKB (http://www.uniprot.org/help/uniprotkb, RRID:SCR_004426) and Uniparc (http://www.uniprot.org/uniparc, RRID:SCR_005818). Chosen sequences were aligned with MAFFT (http://mafft.cbrc.jp/alignment/server, RRID:SCR_011811) and then visualized with the MVIEW tool (https://desmid.github.io/mview, RRID:SCR_024129). Initially, 156 orthologous sequences that shared 50% sequence conservation with Q8NES3 (LFNG, Homo sapiens) were compared (Uniref50 [http://www.uniprot.org/help/uniref, RRID:SCR_010646]; Uniparc) from which six LFNG sequences from human, cow, mouse, rat, African clawed frog, Xenopus, and zebrafish were selected to consolidate orthologous comparison (Uniref50; UniprotKB/Swiss‐Prot [https://www.expasy.org/resources/uniprotkb‐swiss‐prot, RRID:SCR_021164]). Furthermore, four isoform sequences of human LFNG, two isoform sequences of human MFNG, and the sequence for human RFNG were used in the paralogous analysis (Uniprotkb/swiss‐prot). The aforementioned sequences were labeled with their corresponding accession codes accordingly (Figure S2A).

### 2.6 LFNG‐HA western blotting

The protocol for western blotting is reported elsewhere.^24^ Briefly, 15 μL of 1 μg/μL protein lysate (15 μg total protein per lane) was run on an SDS‐polyacrylamide gel and blotted on nitrocellulose membrane. Membranes were blocked for 1 h and incubated overnight with mouse anti‐β‐actin (Santa Cruz, Freemont, CA, USA, Cat: 47778, RRID:AB_2714189; 1:1000) or rabbit anti‐HA (Cell Signaling Technology, Danvers, MA, USA, Cat: 3724, RRID:AB_1549585, 1:1000). The following morning, blots were incubated for 1 h in HRP‐linked goat anti‐mouse IgG (Cell Signaling Technology, Cat: 7076, RRID:AB_330924, 1:1000) or HRP‐linked goat anti‐rabbit IgG (Cell Signaling Technology, Cat: 7074, RRID:AB_2099233, 1:1000) and imaged on a ChemiDoc MP Imaging System (BioRad, Mississauga, ON, CAN, Cat: 12003154). Densitometry was performed in BioRad Image Lab 6.1 Software (http://www.bio‐rad.com/en‐us/sku/1709690‐image‐lab‐software, RRID:SCR_014210).

### 2.7 LFNG‐HA immunofluorescence microscopy

The protocol for immunofluorescence microscopy is reported elsewhere.^24^ Briefly, NIH‐3T3 cells were transiently transfected with each of the *LFNG‐HA* plasmids and incubated for 20 h. The following day, the cells were fixed, permeabilized, blocked, and incubated with rabbit anti‐HA (Cell Signaling Technology, Cat: 3724, RRID:AB_1549585, 1:800) and mouse anti‐Golgi matrix protein 130 (GM130) (BD Biosciences, Mississauga, ON, CAN, Cat: 610822, RRID:AB_398141, 1:800) primary antibodies. Cells were then treated with goat anti‐mouse Alexa Fluor 594 (Thermo Fisher Scientific, Cat: A‐11005, RRID:AB_2534073, 1:500), and goat anti‐rabbit Alexa Fluor 488 (Thermo Fisher Scientific Cat: A‐11034, RRID:AB_2576217, 1:500), followed by HOECHST 3342 (Thermo Fisher Scientific, Cat: H‐3570, 1 μg/mL). Slides were mounted before imaging at 60X on the WaveFX Spinning Disk Confocal Microscope (Quorum Technologies, Cambridge, ON, CAN) at the University of Alberta Cell Imaging Core.

### 2.8 Functional analysis and 3XFLAG‐LFNG western blotting

The protocol for functional analysis is reported elsewhere^22^, ^23^, ^24^ and the description of the method partly replicates the wording. 5 μg of *3XFLAG‐LFNG* plasmid was transfected into COS‐7 cells (~80% confluency) on 100‐mm culture dish using Lipofectamine 3000 (Thermo Fisher Scientific, Cat: L3000001) according to the manufacturer's instructions. Three days after transfection, the protein was isolated, incubated with 10 μL of anti‐DYKDDDDK agarose resin (FujiFilm Wako, Cat: 012–22 781), eluted at 95°C with SDS buffer, then western blotted. The membrane was then incubated overnight at 4°C with anti‐DYKDDDDK antibody (FujiFilm Wako, Cat: 018–22 381, RRID:AB_10659453). The bound antibody was detected the next morning with IRDye 680RD donkey anti‐mouse IgG (Li‐Cor, Lincoln, NA, USA, Cat: 926–68 072, RRID:AB_10953628) on an Amersham ImageQuant Fluor 800 (Cytiva, Tokyo, JPN, Cat: 29399484). The amount of recombinant 3XFLAG‐LFNG protein was estimated with a standard curve of 3XFLAG‐tagged Bacterial Alkaline Phosphatase with Image Quant (GE Life Sciences, Chicago, IL, RRID:SCR_014246).

The Glc‐NAc‐transferase assay mixture contained 10 μL of enzyme‐bound anti‐FLAG affinity resins (100 ~ 200 ng protein of the recombinant LFNG), 50 mM 2‐(N‐morpholino)‐ethanesulfonic acid‐NaOH (pH 6.5) (Nacalai tesque, Kyoto, JPN, Cat: 21623–26), 10 mM MnCl^2^ (Nacalai tesque, Cat: 21211‐45), 0.1 mM UDP‐GlcNAc (Promega, Cat: V7071) as the sugar donor substrate, and 1 mM *p*‐nitrophenyl‐α‐L‐fucose (pNP‐Fuc; Sigma, Cat: N3628) as the sugar acceptor in a total volume of 50 μL. The reaction mixture was incubated at 37°C for 4 h. The UDP reaction product released from UDP‐GlcNAc was mixed with UDP detection reagent from the UDP‐Glo Glycosyltransferase Assay (Promega, Cat: V6961). The newly synthesized ATP was measured using a luciferase/luciferin reaction, and the luminescent signal was detected using an EnSpire multiwell plate reader (PerkinElmer, Waltham, MA, USA, Cat: 6057420).

### 2.9 Statistical analysis

One‐way ANOVA was performed to determine whether a statistically significant difference between three or more groups exists (*p* = 0.05). Upon validation, Bonferroni‐adjusted post hoc analysis was employed to quantify differences between specific groups. Data analyses were performed in Excel (Microsoft, Redmond, WA, USA, https://www.microsoft.com/en‐gb, RRID:SCR_016137) and GraphPad Prism version 10.0.0 for Windows (GraphPad Software, Boston, MA, USA, https://www.graphpad.com, RRID:SCR_002798).

## 3 RESULTS

### 3.1 *LFNG* variants lead to inhibition of protein processing

Protein processing is a critical aspect of LFNG post‐translational regulation during somitogenesis. Improper processing is associated with SCD3‐like phenotype in murine models,^18^ but only one human variant (c.564C>A [p.F188L]) was previously shown to inhibit this process.^20^, ^24^ To test protein processing for each of the nine variants, western blotting was undertaken with cell lysate obtained from NIH‐3T3 cells transiently transfected with variant‐induced HA‐tagged *LFNG* plasmids (Figure 1). Two of the nine variants were previously shown to process normally (p.R174H, p.G256S) and were used as positive controls.^24^ One of the nine variants, p.F188L, was shown to inhibit processing and was used as a negative control^20^, ^24^. In the six experimental conditions, there were variable yet statistically insignificant differences between the amount of unprocessed variant LFNG and WT (MW = 43 kDa) (Figure 1A,B). The three control conditions (p.R174H, p.F188L (see, “Limitations”), and p.G256S) also did not yield significant differences in unprocessed protein. Interestingly, five variants lead to decreased cleavage of LFNG (MW = 34 kDa) compared with WT: p.T149I (*p* = 5.0 × 10^−8^), p.L156R (*p* = 5.8 × 10^−5^), p.W195R (*p* = 9.2 × 10^−10^), p.D201N (*p* = 1.7 × 10^−5^), and p.T281K (*p* = 6.3 × 10^−5^) (Figure 1C,D). No significant difference was observed between p.R286W and WT (*p* = 1.9 × 10^−2^) as the Bonferroni‐adjusted *α* = 5.56 × 10^−3^ (Figure 1D). The amount of the p.F188L control was significantly decreased, as previously reported (*p* = 2.4 × 10^−8^), whereas the differences between the p.R174H and p.G256S controls were not statistically significant (Figure 1C,D). Therefore, the data indicated that only three (p.R174H, p.G256S, and p.R286W) of the nine LFNG variants were normally processed.

**FIGURE 1:**
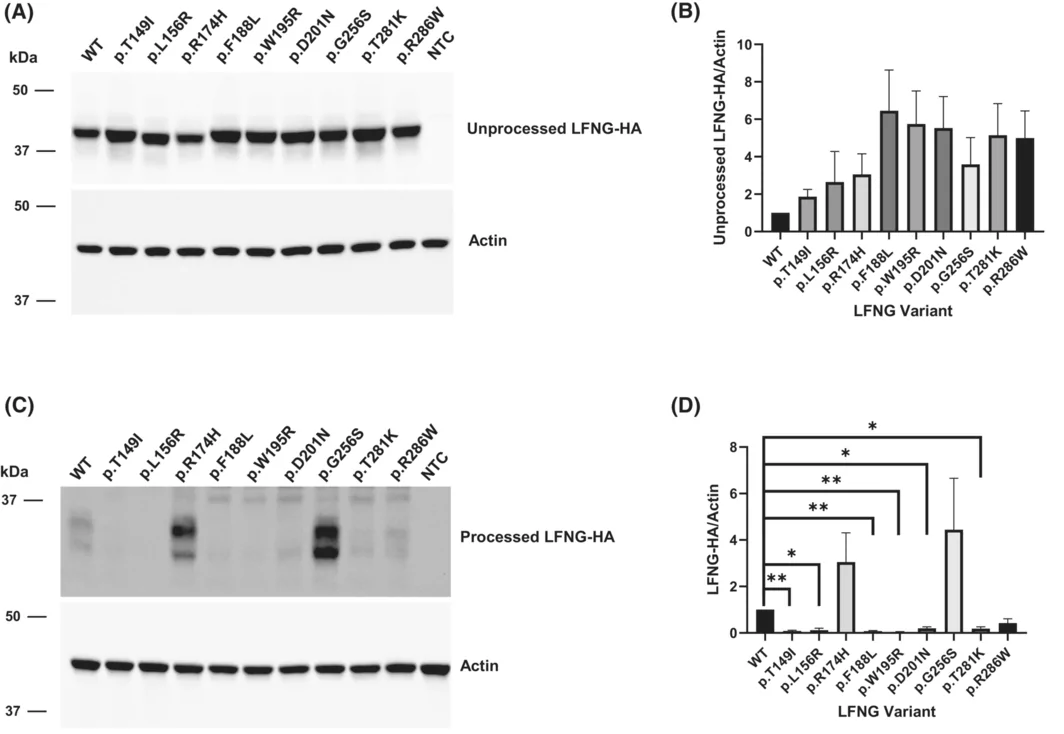
Western blot of LFNG variants to assess protein processing. (A and C) Western blot of cell lysate (15 μg per sample) from NIH‐3T3 cells transiently transfected with *LFNG‐HA* constructs. Samples were run on a 10% SDS gel and membranes were incubated with anti‐HA primary then anti‐Rabbit IgG secondary antibodies or anti‐Actin primary then anti‐Mouse IgG secondary antibodies. (A and C) Representative samples from two separate blots. (B and D) Densitometry of pre‐pro‐LFNG‐HA (B) or processed (D) LFNG‐HA bands normalized to actin. (B) One‐Way ANOVA failed to identify significant differences in the pre‐pro‐LFNG‐HA condition (*F* (4, 9) = 1.54, *p* = 1.8 × 10^−1^). (D) One‐way ANOVA identified significant differences in the LFNG‐HA (*F* (4, 9) = 3.6, *p* = 3.9 × 10^−3^) condition. After Bonferroni post hoc adjustment (*α* = 5.56 × 10^−3^), WT band signal was significantly more intense than those of p.T149I, p.L156R, p.F188L, p.W195R, p.D201N, and p.T281K. Bars indicate mean ± SE of four independent experiments (*N* = 4) plated in triplicate. *p < 1.0 × 10^−4^, **p < 1.0 × 10^−7^.

### 3.2 *LFNG* variants which were not processed were also mislocalized

The variants p.R174H and p.G256S have previously been shown to be associated with normal levels of processed LFNG and colocalization within the Golgi.^24^ Moreover, it was known that p.F188L does not process LFNG appropriately and is mislocalized.^20^, ^24^ As a result, it was hypothesized that protein misplacement inhibits LFNG processing, primarily because SPC6 cleaves LFNG within the Golgi. To test this hypothesis, we predicted that the unprocessed variants (p.T149I, p.L156R, p.W195R, p.D201N, and p.T281K) would be mislocalized whereas the processed variant (p.R286W) would be colocalized with the Golgi when visualized using immunofluorescent microscopy. Colocalization generally appears as a concentrated, overlapping LFNG‐Golgi signal with low levels of diffusive intracellular LFNG signal.^17^, ^20^, ^24^ In contrast, mislocalization tends to present as a diffuse intracellular LFNG signal with slight artifactual or no Golgi signal overlap.

In this experiment, p.F188L was employed as a negative control (Figure 2E) whereas p.R174H (Figure 2D) and p.G256S (Figure 2H) were used as positive, localized controls. As has previously been shown, p.R174H and pG256S, as well as the WT (Figure 2A) control localized appropriately while p.F188L (Figure 2E) did not.^20^, ^24^ Five of the remaining six variants, p.T149I (Figure 2B), p.L156R (Figure 2C), p.W195R (Figure 2F), p.D201N (Figure 2G), and p.T281K (Figure 2I), mislocalized. Notably, in the ninth variant, p.R286W, only some of the cells (Figure 2J,K) were appropriately localized but did so within a “mislocalized” pattern, although this result correlated with relative protein processing (see, “Mislocalization Prevents Processing and Secretion”). Our prediction that the abnormal processing of the variants was due to mislocalization, and was largely supported. Full composites with unmerged images are available in the supplementary information (Figure S1).

**FIGURE 2:**
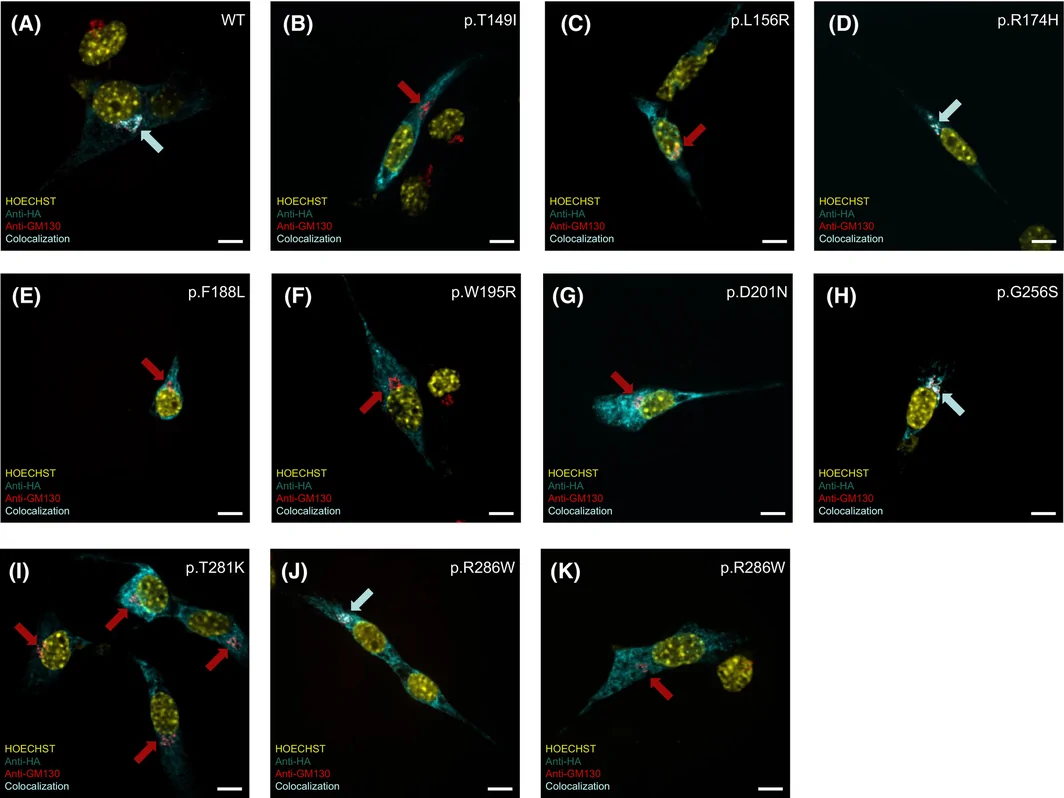
Immunofluorescent imaging of LFNG variants. Immunofluorescent microscopy images of NIH‐3T3 cells transiently transfected with *LFNG* variant plasmids. Cells were treated with rabbit anti‐HA and mouse anti‐GM130 primary antibodies, then fluorescent secondary antibodies followed by HOECHST. Turquoise arrows indicate Anti‐HA/Anti‐GM130 signal colocalization. Red arrows indicate Anti‐GM130 signal and thus lack of Anti‐HA/Anti‐GM130 colocalization. Images are representative samples of 40 cells screened at random across the three independent experiments (*N* = 3), and values (/40) indicate the number of cells which showed the pattern exemplified in the panel. (A) WT‐ (40/40), (B) p.T149I (38/40), (C) p.L156R (36/40), (D) p.R174H (40/40), (E) p.F188L (39/40), (F) p.W195R (38/40), (G) p.D201N (37/40), (H) p.G256S (40/40), (I) p.T281K (38/40), and p.R286W (J and K). Panel (J) shows an example of localization (25/40) whereas panel (K) shows mislocalization for the same variant (15/40). Scale bars = 15 μm. The fluorescent signal was artificially altered for color‐blind accessibility.

### 3.3 The p.T149I substitution is functionally hypomorphic whereas p.W195R and p.T281K are null

Although most of the variants in this study have been previously assessed for glycosyltransferase activity, p.T149I, p.W195R, and p.T281K have not been assessed. We therefore quantitatively assessed the effect of these three variants on Glc‐NAc‐transferase activity through the use of a previously validated assay.^22^, ^23^ Only variants which were not previously assessed were tested here as the quantitative nature of this assay consistently demonstrates significant evidence of reliability in previous work^22^, ^23^, ^24^ (Figure 3 Legend; Figure 3). To this end, we employed a *LFNG* plasmid that encoded the soluble FRINGE domain of LFNG with an N‐terminal Mouse Anionic Trypsin 2 (PRRS1) pre‐pro‐trypsin (PPT) leader sequence and 3XFLAG epitope tag in place of the LFNG transmembrane domain and signal peptide.^23^ This PPT (MSALLILALVGAAVA) was shown to direct 3XFLAG‐LFNG to the secretory pathway via the Golgi to allow for highly efficient isolation.

**FIGURE 3:**
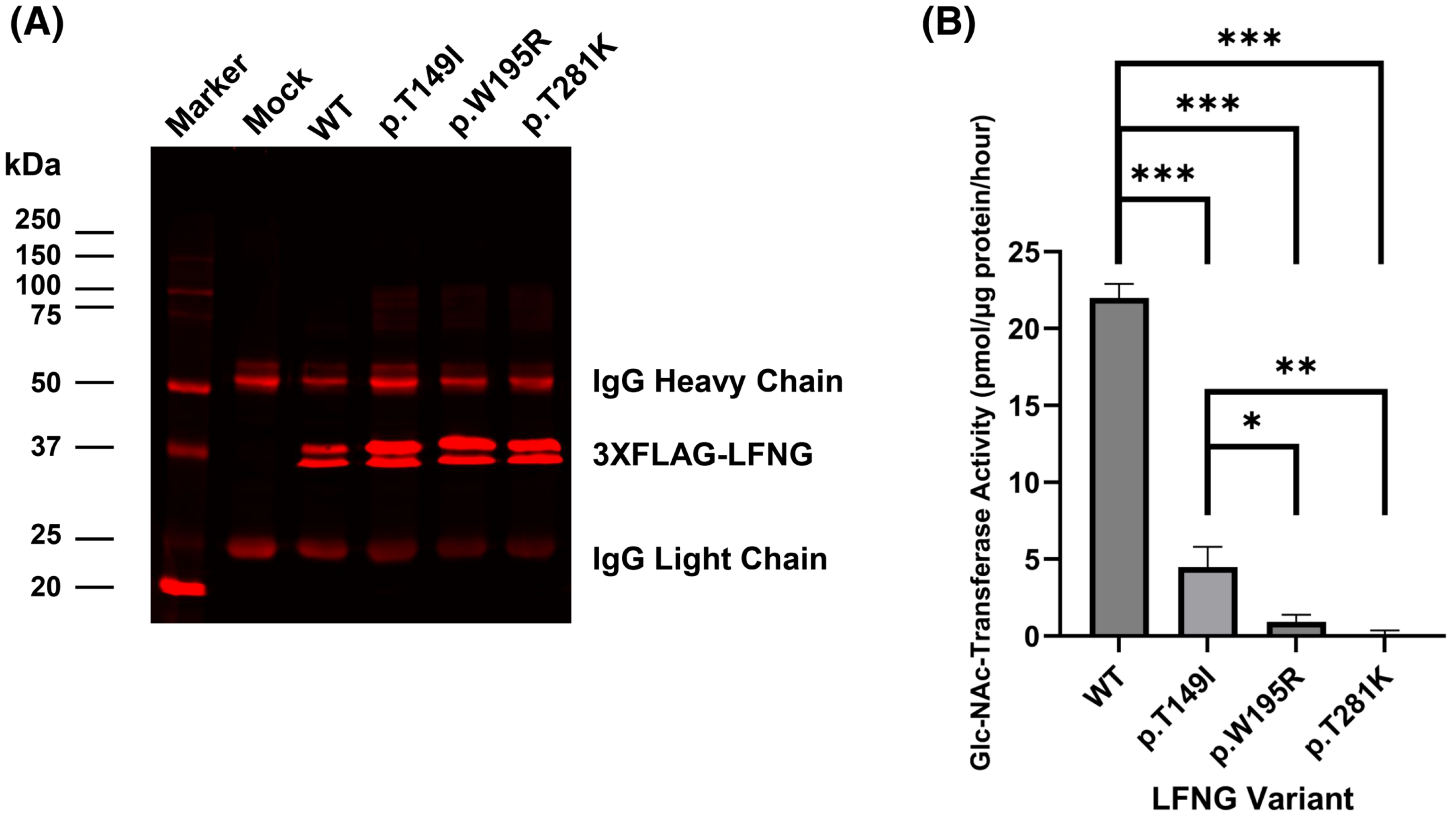
Quantitative analysis of LFNG glycosyltransferase activity. (A) Fluorescent western blotting of 3XFLAG‐tagged WT, p.T149I, p.W195R, and p.T281K‐LFNG proteins. The expression level of recombinant 3XFLAG‐LFNG was quantified by the fluorescent intensity using a calibration curve of 3XFLAG‐bacterial alkaline phosphatase (Sigma). Two bands of ~25 and ~50 kDa were light and heavy chains of IgG, respectively, derived from anti‐FLAG antibody‐conjugated affinity gel, which was utilized for the purification of recombinant LFNG. (B) Glc‐NAc‐transferase activity of the recombinant LFNG. The recombinant LFNGs were incubated with UDP‐GlcNAc and pNP‐Fuc as the donor and acceptor substrates, respectively. The amount of reaction product, UDP, was determined by UDP‐Glo™ Glycosyltransferase Assay kit. The variant LFNGs showed significantly reduced enzyme activity. *p < 5.0 × 10^−2^, **p < 1.0 × 10^−2^, ***p < 1.0 × 10^−10^ vs. WT enzyme by ANOVA and Bonferroni post hoc comparisons (*N* = 6).

To determine whether 3XFLAG‐LFNG was secreted, a western blot of protein lysate and media from COS7 cells transiently transfected with each of the *LFNG* variants was undertaken. p.T149I, p.W195R, and p.T281K 3XFLAG‐LFNG variant constructs were not detected in the media (data not shown; Table 2) but all were present in the cell lysate (Figure 3A). Therefore, only LFNG from cell lysate was isolated for further analysis. Enzyme activity was assessed using the previously reported UDP‐Glo assay.^22^, ^23^ Briefly, resin‐bound 3XFLAG‐LFNG was reacted at 37°C for 4 h in the presence of UDP‐GlcNAc (the donor) and *p*‐nitrophenyl‐α‐L‐fucose (the acceptor). The reaction by‐product (UDP) was then employed in a luminescent reaction as a surrogate to quantitatively measure enzyme activity. Therefore, decreased luminescence indicated that the variant had a more deleterious effect on enzyme activity. The data indicated that p.T149I (*p* = 2.1 × 10^−11^), p.W195R (*p* = 6.8 × 10^−13^), and p.T281K (*p* = 3.2 × 10^−13^) had significantly decreased enzymatic activity (Figure 3B). Although there was no statistically significant difference in activity between p.W195R and p.T281K (*p* > 9.9 × 10^−1^), p.T149I activity was significantly elevated compared with both p.W195R (*p* = 4.3 × 10^−2^) and p.T281K (*p* = 8.5 × 10^−3^) (Figure 3B). p.T149I is the second *LFNG* allele associated with SCD3 to be described with hypomorphic activity.

### 3.4 In silico proteomic and genetic analysis partially explains functional perturbations

To further investigate a potential biochemical mechanism to explain the changes in p.T149I, p.L156R, p.W195R, p.D201N, p.T281K, and p.R286W LFNG variant enzyme, we modeled the variants in silico with AlphaFold2 generated structures. The structures of p.F188L, p.R174H, and p.G256S were assessed elsewhere.^20^, ^24^ The p.T149I substitution (RMSD = 0.542) appeared to have led to the disruption of a hydrogen bond between Thr‐149 and His‐126 and destabilized a region containing two alpha helices between Asp‐131 and His‐159 (Figure 4A) (Table 2). Similarly, the p.L156R (RMSD = 0.521) substitution shifted the alpha helix from Asp‐153 toward Thr‐160 (Figure 4B). The change induced by p.W195R (RMSD = 0.392) shifted His‐219 outwards from the protein core and was predicted to destabilize the Arg‐195/Ser‐216 loop but not any flanking secondary structures (Figure 4C). Finally, p.T281K (RMSD = 0.476) pushed against Arg‐284 and Tyr‐295 and appears to have pivoted an entire beta‐hairpin from residues 234–250 away from the protein core (shifted up to a maximum of 2.6 angstroms) (Figure 4D). Both p.D201N (RMSD = 0.318) and p.R286W (0.285) showed no significant predicted structural changes (Data not shown).

**FIGURE 4:**
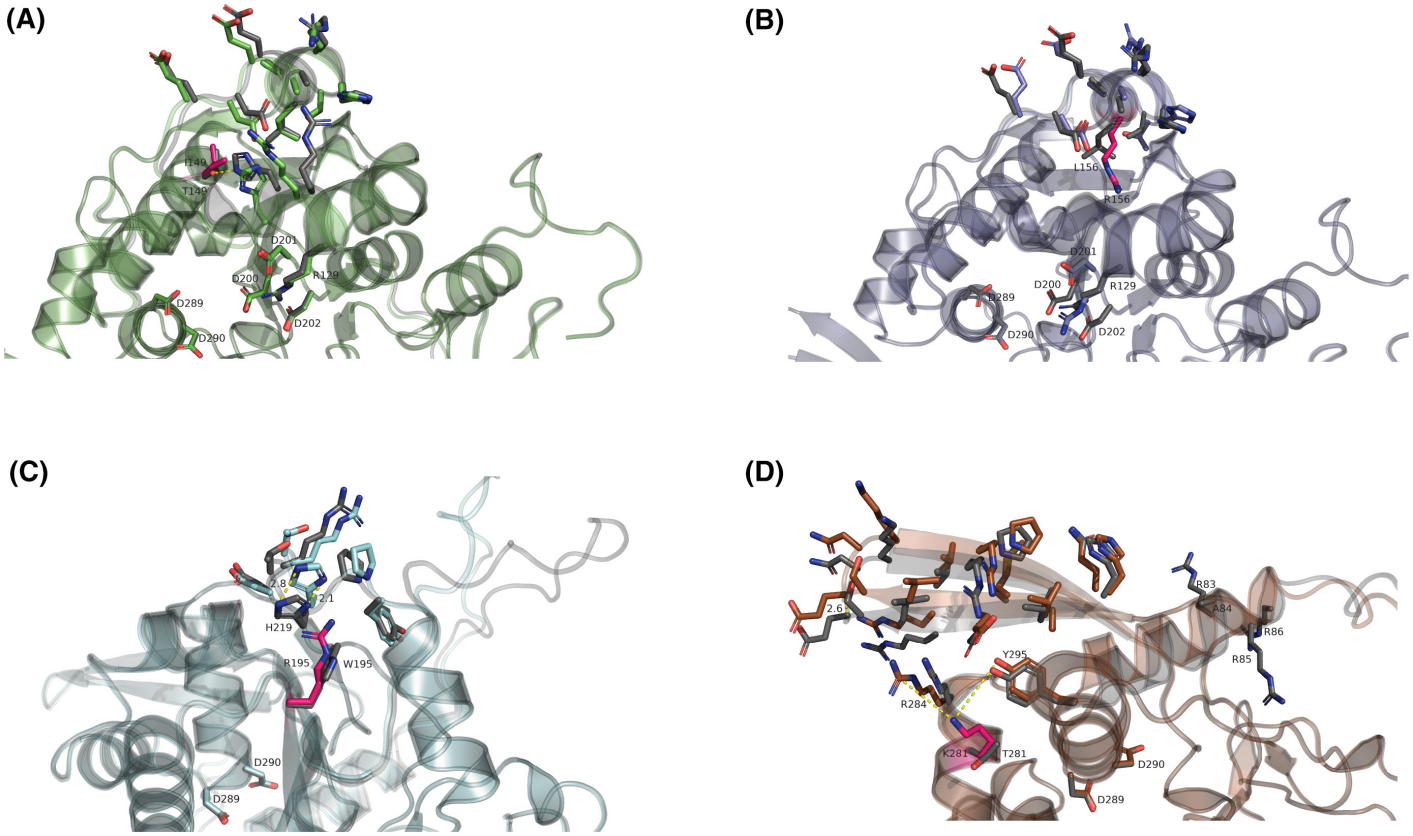
In silico construction of LFNG variants. Overlayed models of WT (gray) and variant (colored) LFNG tertiary structures. In all cases, the referenced secondary structures are outlined with enlarged side chains. (A) p.T149I disrupts a hydrogen bond between p.H126, leading to the destabilization of a region encompassing two alpha helices (residues p.D131 to p.H159). Variant structure overlayed in green. (B) p.L156R similarly affects this region but primarily shifts the alpha helix from p.D153 to p.T160. Variant structure overlayed in purple. (C) p.W195R induces a shift in p.H219 outwards from the protein core, predicted to destabilize a loop (p.R195 to p.S216) without impacting flanking secondary structures. Variant structure overlayed in turquoise. (D) p.T281K exerts pressure on p.R284 and p.Y295, leading to the predicted pivoting of an entire beta‐hairpin (residues 234–250) away from the protein core, with each position shifted up to a maximum of 2.6 angstroms. Variant structure overlayed in orange. Variants p.D201N and p.R286W show no significant predicted structural changes (Data not shown). Critical residues: Active site, p.D290. DxD motif (UDP‐GlcNAc/Mn^2+^ binding), p.D200‐p.D201‐p.D202. Mn^2+^ binding, p.H314.

Previous work has demonstrated evidence suggesting substitutions closer to the active site are more likely to affect catalysis directly whereas those farther may affect catalysis through structural changes.^29^ This would predict that substitutions closer to key enzymatic sites would be less likely to affect structure and more likely to affect function. Therefore, we measured α‐carbon to α‐carbon distances between each variant's substituted amino acid and LFNG's key enzymatic residues, the DxD motif (p.D200‐D201‐D202), and the catalytic residue (p.D290) (Table 2). In order of closest to farthest, the predicted α‐carbon to α‐carbon distances from p.D20 to each variant were as follows: (1) p.D201 = 0.0 Å, (2) p.G256 = 8.6 Å. (3) p.T149 = 10.0 Å; (4) p.L156 = 16.3 Å; (5) p.R174 = 17.4 Å; (6) p.F188 = 17.8 Å; (7) p.W195 = 19.0 Å; (8) p.R286 = 24.2 Å; (9) p.T281 = 24.5 Å. In order of closest to farthest, the predicted α‐carbon to α‐carbon distances from p.D290 to each variant were as follows: (1) p.G256 = 8.7 Å, (2) p.R174 = 10.1 Å. (3) p.R286 = 11.7 Å; (4) p.R281 = 11.8 Å; (5) p.D201 = 15.3 Å; (6) p.F188 = 16.1 Å; (7) p.W195 = 16.8 Å; (8) p.T149 = 17.3 Å; (9) p.L156 = 26.1 Å.

To begin assessing the evolutionary importance of each residue, conservation was estimated with orthologous/paralogous analysis. Across 162 LFNG orthologs, all sequences associated with the substitutions were highly conserved (Figure S2). Specifically, p.T149 (GERP = 3.95), p.L156 (GERP = 4.83), and p.W195 (GERP = 5.32) were ~ 90% conserved with no variation in amino acid residue (Uniref50; Figure S2B). Residues p.D201 (GERP = 5.32), p.T281 (GERP = 4.70), and p.R286 (GERP = 4.70) were ~ 100% conserved (Uniref50; Figure S2B). To further examine the hypomorphic p.T149I substitution, we assessed the paralogous conservation of Thr‐149. Similar to Arg‐174, Thr‐149 was only ~80% conserved across three known fringe proteins (LFNG, RFNG, and MFNG) and their associated isoforms (Figure S2C).

Finally, REVEL scores were obtained as a quantifiable metric of predicted pathogenicity (Table 2). REVEL scores range from 0.0 (benign) to 1.0 (pathogenic) and are an aggregate of 13 predictive variant pathogenicity algorithms.^30^ In order of most predicted pathogenicity to least, the scores were as follows: (1) p.W195R = 0.9; (2) p.G256S = 0.89; (3) p.D201N = 0.84; (4) p.L156R = 0.74; (5) p.R286W = 0.73; (6) p.T149I = 0.68; (7) p.T281K = 0.62; (8) p.R174H = 0.52; (9) p.F188L = 0.51.

## 4 DISCUSSION

SCD is a monogenic condition caused by loss‐of‐function variants in six genes critical to somitogenesis: *Delta‐like Ligand 3* (*DLL3)* (SCD1), *Mesoderm Posterior Protein 2* (*MESP2)* (SCD2), *LFNG* (SCD3), *Hairy Enhancer of Split 7* (*HES7)* (SCD4), *T‐box Transcription Factor 6* (*TBX6)* (SCD5), and *RIPPLY2* (SCD6).^9^ Although subtle phenotypic variation exists between subtypes, variation within subtypes remains an area of minimal investigation. Genotype–phenotype correlations could lead to more accurate genetic counseling about prognosis and clinical management plans. Assessing the possibility of genotype‐dependant phenotypic variance in SCD3 is particularly useful as *LFNG* variants can lead to diverse levels of functional inhibition.^24^, ^29^ Therefore, we sought to investigate the functional effect of nine *LFNG* variants which cause SCD3 in the hopes of more deeply understanding the genotype–phenotype relationship. All variants except for c.766G>A (p.G256S) and c.521G>A (p.R174H) are misprocessed and mislocalized, and all variants except for c.521G>A (p.R174H) and c.446C>T (p.T149I) are enzymatically inactive (review Tables 1 and 2). The American College of Medical Genetics and Genomics (ACMG) uses strict criteria to classify a variant as pathogenic.^31^, ^32^ These data satisfy PS3 (functional evidence via well‐established functional assay) criteria for the pathogenicity of c.583 T>C (p.W195R), c.842C>A (p.T281K), and c.446C>T (p.T149I), and further validates this distinction for the remaining six variants.

### 4.1 Mislocalization is a common etiology of SCD3

In order for LFNG to transfer GlcNAc to NOTCH receptors it must be located within the Golgi lumen.^1^, ^13^, ^20^ Here, it acts alongside other critical glycosylating enzymes to potentiate appropriate NOTCH receptor–ligand interactions. Without LFNG, the “wrong” receptor–ligand interactions ensue, and the segmentation clock fails to oscillate.^4^, ^10^, ^11^, ^33^ Therefore, the current understanding suggests that genotype may not explain phenotypic variability between SCD3 cases caused by the equally null, mislocalized variants.

As mislocalization is suspected to necessarily perturb LFNG activity, and thus cause the SCD3 phenotype, it is important to explore factors which might influence a variant's effect on subcellular localization. One well‐known cause of mislocalization is variant‐induced structural perturbation (see “Future Directions”). Previous work has suggested that variants distal to LFNG's catalytic site (p.D290) or Mn^2+^ binding residues (DxD, p.D200‐p.D201‐p.D202) are more likely to structurally alter LFNG compared with those which are proximal.^29^ In support of this, the three variants closest to the catalytic site (1) p.G256S = 8.7 Å; (2) p.R174 = 10.1 Å; (3) p.R286 = 11.7 Å were localized (p.G256S), localized (p.R174H), and partially localized (p.R286W) (Figures 2 and 3; Table 2). Furthermore, each of these localized variants were not predicted to affect the structure whereas four of the six mislocalized variants were. Two variants which fail to support this notion include p.F188L and p.D201N. Although both variants were farther from the catalytic site, they were not predicted to affect structure and failed to colocalize with the Golgi (Figure 2; Table 2).^20^, ^24^ There also does not appear to be a correlation between proximity to DxD and localization (Table 2). Critically, the unique biochemical properties of each variant confound the suggestion that variant proximity to the catalytic site is alone responsible for mislocalization; rather, it is likely to be a single contributing factor among many. Precise structural location, substitution, and local side‐chain interactions, discussed at length here and elsewhere, play very significant modulatory roles.^20^, ^22^, ^23^, ^24^, ^25^, ^29^ Finally, the inherent predictive limitations of AlphaFold and in silico software further limit the strength of this influence. Therefore, although promising, it remains unclear whether proximity to these key sites, structural disturbance, and mislocalization are linked. It is hoped that future investigations can utilize the in vitro data generated here to assess the possibility of such a contributing factor more thoroughly.

### 4.2 Mislocalization prevents processing and secretion

The examination of protein processing is essential in understanding the impact of LFNG variants on the modulation of SCD3 presentation. This significance is underscored by the fact that impaired protein processing has been associated with the SCD3 phenotype in mouse models.^17^, ^18^ Nonetheless, earlier studies have indicated that aberrant processing might result from the protein being misplaced to an incorrect location, rather than being a separate occurrence^24^. Indeed, data from current work supports this hypothesis as all mislocalized variants were not processed (Figures 1 and 2). The basis for this discovery aligns with evidence showing that LFNG undergoes cleavage by the pre‐pro‐protein convertase SPC6, which is found exclusively within the Golgi apparatus.^17^, ^18^ Additionally, since these variant constructs are not released into the media (Table 2), they could not have entered the Golgi's secretory pathway. This study provides strong support for the notion that the incapacity to reach the Golgi apparatus obstructs the processing and subsequent release of LFNG. Furthermore, it underscores the concept that the FRINGE domain is the fundamental reason behind this unsuccessful translocation, given that the transmembrane domain was deliberately excluded from this experiment (See, “The p.T149I Substitution is Functionally Hypomorphic whereas p.W195R and p.T281K are Null”).^22^, ^23^

Importantly, the results associated with p.R286W provide additional confirmation that mislocalization impedes both protein processing and secretion. In our analysis of p.R286W localization, we noticed distinct LFNG‐Golgi colocalization in some cells (Figure 2J) and clear signs of misplacement in others (Figure 2K). Furthermore, colocalized cells possessed a diffuse LFNG signal characteristic of mislocalized cells (Figure 2B,C,E–G,I,K) in contrast to typical localized conditions here (Figure 2A,D,H) and elsewhere.^17^, ^20^, ^24^ These observations remained consistent across various experimental replicates. As we delved into the analysis of protein processing, it became clear that p.R286W underwent cleavage at a significantly slower pace compared with p.R174H and p.G256S, with roughly 30 times less processed protein (Figure 1D). Evidently, because there was a smaller population of cells expressing properly localized LFNG (Figure 2J,K), a diminished number of cells were able to produce cleaved protein, which explains both sets of findings (Figure 1D). These findings harmonize with previous research that suggested p.R286W was secreted with both the artificial and endogenous signal peptides.^23^ In this study, the prolonged multi‐day incubation period would have allowed the partially functional localization mechanism enough time to release an adequate quantity of LFNG into the culture medium. However, the mechanism by which partial localization occurs remains unexplained. The possibility of plasmid diversity can be reasonably dismissed, as all plasmid samples underwent sequencing and validation before transfection. Additionally, disproportional variations in cell‐to‐cell transfection efficiencies are unlikely to be the root cause, as all other experimental conditions yielded consisten results across all replicates. Finally, although Z‐stacking was unable to completely prevent artifactual colocalization, p.R286W had far more cells associated with both patterns and this overlap did not appear artifactual in origin (Figure 2 legend; Figure S1).

In silico, the distinctive patterns observed in p.R286W hint at nuanced spatial dynamics in a potentially unrecognized region. Examination of the p.R286W variant reveals that it has remarkable proximity to variants p.T281K and p.R174H. Unlike p.T281K, which exhibits complete mislocalization, and p.R174H, which localizes to the Golgi, p.R286W presents a partial colocalization pattern in some cells (Figure 2J) alongside clear displacement in others (Figure 2K). Examining this ambiguous region in more detail may aid in elucidating residues or domains critical to the intracellular translocation of LFNG.

### 4.3 Residual glycosyltransferase activity of p.T149I does not modulate phenotype

Previous research has primarily concentrated on Glc‐NAc‐transferase activity when evaluating the functional impact of *LFNG* variants.^20^, ^22^, ^23^ This is because, without glycosyltransferase activity, LFNG cannot exert an effect on NOTCH receptors, and consequently, it cannot influence the segmentation clock. We report the sixth and seventh variants associated with complete nullification of LFNG enzymatic activity (c.583 T>C [p.W195R]; c.842C>A [p.T281K]) (Figure 3; Table 2). Biochemically, these variants are predicted to exhibit significant structural changes. Protein modeling suggests that p.W195R shifts His‐219 outward from the protein core (Figure 4C), whereas in p.T281K, the beta‐hairpin from residues 234–250 undergoes a significant shift (Figure 4D). Both of these changes can reasonably be attributed to the complete nullification of Glc‐NAc‐transferase activity.

Although many variants lead to complete functional knockout (Table 2), previous work has characterized a variant associated with SCD3 with residual Glc‐NAc‐transferase activity (p.R174H).^24^ Here, we report the second partially active variant associated with an SCD3 phenotype, c.446C>T (p.T149I) (Figure 3B; Table 2). Although both variants lead to similar levels of functional activity, they differ in structural perturbation and effect on localization. Primarily, in silico analysis suggest that the variants affect different regions of the enzyme (Figure 4A; Table 2) as structural changes are only associated with p.T149I and not p.R174H. Secondly, unlike p.R174H, p.T149I is mislocalized and therefore would not be able to act on NOTCH receptors with its residual activity. Indeed, the phenotype of the proband with homozygous c.446C>T variants has very few vertebrae (<11) and ribs (<10 bilaterally), significant kyphoscoliosis, and pronounced pebble beach signSub.^25^ This suggests that this patient's phenotype may not have been modulated by residual LFNG activity.

### 4.4 Trunk length may be sensitive to residual LFNG activity

Our findings suggest that six of the seven previously reported probands carry biallelic null *LFNG* variants. Except for p.R174H, all other variants display mislocalization and/or complete loss of enzymatic activity, preventing GlcNAc transfer to NOTCH receptors (See Table 2).^20^ Clinical data is incomplete for the six probands (Table S2),^20^, ^21^, ^22^, ^23^, ^25^ but heights trend below the 5th percentile, and vertebral numbers are typically fewer than 17, with variable yet often severe scoliosis presentation. The seventh proband is compound heterozygous for p.R174H and null p.G256S LFNG alleles, presenting a different axial phenotype with more vertebrae and a longer trunk, perhaps due to relatively increased LFNG activity compared with null genotypes.^24^ Supporting this possibility of genotype‐dependant changes of the axial skeleton, murine models which utilized *LFNG*‐dosage as a proxy for varied LFNG function demonstrated that decreasing dosages correlated with progressively fewer somites and vertebrae.^34^, ^35^, ^36^ However, this hypothesis does not explain additional SCD3 features like arachno/camptodactyly and auditory abnormalities.^20^, ^28^ It also does not account for individual genomic variation which may influence the genotype–phenotype relationship. Future work assessing the impact of *LFNG* variants with different phenotypic manifestations in iPSC‐derived organoid‐development models^5^, ^6^, ^7^ may be able to determine which, if any, factors influence the varied presentation of these additional phenotypes. Correlating this with a quantitative assessment of the variants’ effect on NOTCH activation in isolation with a previously utilized assay^20^ may allow for further elucidation of factors which contribute to the genotype–phenotype relationship. Crucially, more probands will need to be identified to draw any conclusions regarding the expansion of the SCD3 phenotypic spectrum.

### 4.5 Limitations

Primarily, assessing protein processing and localization via transient and unstable transfections introduces internal reliability concerns, particularly evident in the quantity and standard error of intracellular unprocessed LFNG‐HA (Figure 1B). In contrast to previous research, we observed no significant increase in unprocessed protein levels for p.F188L compared with WT (Figure 1A,B).^24^ Processed protein data (Figure 1C,D) was more internally reliable and therefore appeared dependable in determining whether *LFNG* variants inhibit processing. However, statistical variability was still evident in this experiment, particularly in the localized, processed samples (p.R174H and p.G256S), where there may appear to be a qualitative increase in LFNG‐HA compared with WT (Figure 1D). Although not suggested by statistical analysis, future investigations harnessing different methods to further understand LFNG processing should remain open to the possibility of novel, more nuanced mechanisms.

Secondly, this study was unable to provide evidence regarding the particular subcellular compartment(s) to which LFNG is mislocalized. While mislocalization likely hinders LFNG's ability to support somitogenesis, particularly given that each mislocated variant is enzymatically inactive, it remains unclear to what degree an enzymatically active and mislocalized variant would impact somitogenesis.

Thirdly, evaluating LFNG function under standardized conditions ignores Golgi pH variations that can affect LFNG activity.^19^ Since pH variability might influence LFNG glycosyltransferase activity in vivo, investigating whether pH fluctuations partially rescue or further diminish enzyme activity could provide insights into LFNG's Golgi lumen regions that act on NOTCH. This protocol and others^22^, ^23^, ^24^, ^29^ may also cause protein misfolding due to the extended incubations at 37°C (a three‐day expression period followed by either 30‐min reaction or 4‐h reaction). However, previous work did not identify misfolding with other LFNG variants after 72 h and 30 min,^29^ and thus the additional 3 h and 30 min may not substantially impact protein folding. Irrespective of likelihood, future work should be aware of this factor when conducting glycosyltransferase assays and interpreting their results.

Finally, inconsistent phenotypic reporting hampers accurate genotype–phenotype interpretations. Gathering comprehensive clinical data including, but not limited to: age, sex, consanguinity, height (absolute and relative), arm‐span to height ratio, mid‐parental height, vertebral body number, rib number, scoliosis presence/cobb angle, and descriptions of both axial and non‐axial characteristics (Table S2) is vital for understanding the SCD3 genotype–phenotype relationship. To this end, a patient registry can aid data collection, fostering collaboration and a deeper disease understanding, enhancing molecular data interpretation across related studies.

### 4.6 Future directions

A significant gap in LFNG research remains a focused, detailed investigation of the processes which govern its subcellular transport. Although we previously reported that LFNG mislocalizes to the cytoplasm due to its diffuse distributive pattern,^24^ we have been unable to conclusively illustrate the specific subcellular compartment to which the variants mislocalize. An alternative explanation is that variant LFNG is actually mislocalized to the endoplasmic reticulum (ER), as the diffuse pattern is characteristic of ER‐resident proteins (Figure 2B,C,F,G,I–K).^37^, ^38^ One explanatory mechanism could be misfolding in response to amino acid substitutions.^39^ Indeed, previous evidence from disease‐causing variants in *Glycosyltransferase 8 Domain–Containing Protein 1* (*GLT8D1*), a GT‐A fold family, DxD motif‐ containing glycosyltransferase with a type‐II transmembrane domain with similarities to *LFNG*, has shown misfolding and persistence in the ER.^38^ Since misfolding can arise from substitutions in various regions throughout the protein, findings corroborating this hypothesis may explain the wide spatial distribution of variants that result in mislocalization (Table 2).

Future work focusing on the underlying principles and mechanisms of LFNG transport and mislocalization may address this gap and enhance the current understanding of these critical subcellular processes. Such objectives may involve identifying the compartment(s) to which LFNG mislocalizes, exploring the mechanisms and factors involved in LFNG folding, determining the protein(s) responsible for transporting LFNG to the Golgi apparatus (and where these transport proteins bind LFNG) and investigating the potential role of the N‐terminal in translocation. While LFNG mislocalization is recognized as a molecular cause of SCD3, irrespective of its exact location, a more in‐depth inquiry could reveal unidentified factors influencing the SCD3 phenotype.

An overarching difficulty for such a project would be determining the extent to which LFNG misfolding is a consequence of transfection protocol and cell line. To date, transient transfection has been exclusively utilized for the analysis of LFNG localization.^20^, ^24^ This approach may lead to an exaggeration of the misfolding response if the endogenous folding machinery was overwhelmed by transient transection.^40^ Therefore, the discrepancy between genuine and artifactual signals would make it difficult to utilize the findings for genotype–phenotype interpretations and to discriminate between variant‐dependant differences in mislocalization (e.g., p.R286W; Figure 2J,K). Secondly, the protein transport mechanisms, folding capacity, and misfolding responses unique to each cell line could lead to different extents of mislocalization.^31^, ^39^ Although variants appear to display similar patterns in both C2C12 and NIH‐3T3 cells,^20^, ^22^, ^23^, ^24^ quantitative differences have yet to be investigated. Standardization between cell lines could help to increase accuracy and produce more generalizable findings. In light of these potential challenges, we recommend culturing physiologically relevant cell lines, aggregating data from many cell lines and transfection methods (e.g., stable and transient), and/or utilizing CRISPR‐based mutagenesis of endogenous *LFNG* alleles.

## 5 CONCLUSIONS

In this work, we fully or partially characterized the p.T149I, p.L156R, p.W195R, p.D201N, p.T281K, and p.R286W LFNG variants. We also replicated functional analysis for p.R174H, p.F188L, and p.G256S. In this set of samples, we conclude that p.G256S and p.R174H are the only appropriately localized and processed LFNG variants, and p.R174H and p.T149I are the only variants with residual glycosyltransferase activity. We also conclude that protein mislocalization resulting in impaired protein processing and secretion is a common disease mechanism causing SCD3. In silico LFNG variant modeling was unable to conclusively identify a mechanism for this phenomenon, although proximity to the catalytic residue remains promising. Finally, we hypothesize that the axial phenotype of SCD3 may be closely linked with genotype and not other influences. It is hoped that future work will gather updated clinical data from each of the probands and examine *LFNG* variants in the context of iPSC‐derived organoid models. Such work may allow for a more precise investigation of the relationship between SCD3 genotype and phenotype.

## AUTHOR CONTRIBUTIONS

P.W. and P.K. designed the project, F.F. and P.W. performed cloning and subcloning, P.W., F.F., and C.S. performed the western blots, K.S. and P.W. performed the immunofluorescence assays, K.S. imaged the immunofluorescence samples, S.M., S.Y., P.W., and F.F. performed the functional analysis, S.M. and P.W. western blotted the functional samples, A.B. and C.O. compiled and analyzed the in silico protein/genetic analysis, A.B., P.W., S.Y., and S.M. statistically assessed the data, and P.W. and P.K. drafted the manuscript with original scientific insight and revisions contributed equally from all authors.

## DISCLOSURES

The authors report no conflicts of interest.

## ETHICS STATEMENT

This research did require ethical approval as per TCPS‐2 (2022) Article 12.21, the University of Alberta Research Ethics Board.

## Supporting information


Table S1.

Table S2.

Figure S1.

Figure S2.

## Data Availability

The data that support the findings of this study are available in the methods and/or supplementary material of this article. Materials are available upon request to the corresponding author.
