# Supplementary material for: Integrative analysis of Lunatic Fringe variants associated with spondylocostal dysostosis type‐III
Source: FASEB J. 2024 Jun 25;38(13):e23753. doi: 10.1096/fj.202302651RR (PMC13688047; doi:10.1096/fj.202302651RR)
Supplement: Supplementary file 1 [file FSB2-38-e23753-s001.docx]

**Supplemental Materials**

**Figure S1: Composites of Immunofluorescent Imaging of LFNG Variants.** Immunofluorescent microscopy images of NIH-3T3 cells transiently transfected with *LFNG* variant plasmids. Cells were treated with rabbit anti-HA and mouse anti-GM130 primary antibodies, then fluorescent secondary antibodies followed by HOECHST. Turquoise arrows indicate Anti-HA/Anti-GM130 signal colocalization. Red arrows indicate Anti-GM130 signal and thus lack of Anti-HA/Anti-GM130 colocalization. Images are representative samples of 40 cells screened at random across the three independent experiments (N = 3), and values (/40) indicate number of cells which showed the pattern exemplified in the panel. A) WT- (40/40), B) p.T149I (38/40), C) p.L156R (36/40), D) p.R174H (40/40), E) p.F188L (39/40), F) p.W195R (38/40), G) p.D201N (37/40), H) p.G256S (40/40), I) p.T281K (38/40), and p.R286W (J, K). Panel J) shows an example of localization (25/40) whereas panel K) shows mislocalization for the same variant (15/40). Scale bars = 15 µm.
