# Supplementary material for: Integrative analysis of Lunatic Fringe variants associated with spondylocostal dysostosis type‐III
Source: FASEB J. 2024 Jun 25;38(13):e23753. doi: 10.1096/fj.202302651RR (PMC13688047; doi:10.1096/fj.202302651RR)
Supplement: Supplementary file 2 [file FSB2-38-e23753-s002.docx]

**Supplemental Materials**

| *LFNG* Variant | Forward (5’ to 3’) | Reverse (5’ to 3’) |
| --- | --- | --- |
| c.446C>T | TCTTCCCCGTCAATGAAGATGAACGTCATCTCCTTG | CAAGGAGATGACGTTCATCTTCATTGACGGGGAAGA |
| c.467T>G | CGTGTGCCTGGCCCGGGCCTCATCTTC | GAAGATGAGGCCCGGGCCAGGCACACG |
| c.583T>C | ACGTGGCAGAACCGCTTCCTGCCGGAC | GTCCGGCAGGAAGCGGTTCTGCCACGT |
| c.601G>A | GTTGACGTAGTTGTCATTGTCCACGTGGCAGAACC | GGTTCTGCCACGTGGACAATGACAACTACGTCAAC |
| c.842C>A | CCGCTCAGCCTTATTCATGAAGTGACCCCC | GGGGGTCACTTCATGAATAAGGCTGAGCGG |
| c.856C>T | TCATCAGGCAGCCAGATCCGCTCAGCC | GGCTGAGCGGATCTGGCTGCCTGATGA |

Table S1: Primers for Site-Directed Mutagenesis.
