# Supplementary material for: Integrative analysis of Lunatic Fringe variants associated with spondylocostal dysostosis type‐III
Source: FASEB J. 2024 Jun 25;38(13):e23753. doi: 10.1096/fj.202302651RR (PMC13688047; doi:10.1096/fj.202302651RR)
Supplement: Supplementary file 3 [file FSB2-38-e23753-s003.docx]

| Additional Findings Description | Discussed, present | Not Discussed | Not Discussed | Discussed, none | Discussed, none | Discussed, present | Discussed, none |
| --- | --- | --- | --- | --- | --- | --- | --- |
| Axial Abnormally Description | Discussed | Minimally Discussed | Minimally Discussed | Discussed | Discussed | Minimally Discussed | Discussed |
| Scoliosis/  Cobb Angle | Scoliosis/ N/A | N/A†/  N/A | N/A†/  N/A | Scoliosis/  70° (T9-L3) | No scoliosis | Scoliosis, N/A | Scoliosis/  38° (L1-S1) |
| Rib Number | N/A† | N/A | N/A | N/A† | N/A† | N/A† | 10 left,  9 right |
| Vertebral Body Number | N/A† | N/A | N/A | N/A† | N/A† | N/A† | >20 |
| Mid-Parental Height | N/A | N/A | N/A | N/A | N/A | N/A | 179 cm |
| AS:H Ratio | 1.20 | N/A | N/A | N/A | N/A | N/A | 1.08 |
| Height/ SD | 155cm/ N/A | N/A/  N/A | N/A/  N/A | N/A/  -2.1 SD | N/A/  -2.5 SD | N/A/  N/A | N/A/  -0.67 to  -1.28 SD |
| Consanguinity | N/A | No | No | No | No | No | No |
| Sex | M | N/A | N/A | M | M | N/A | M |
| Age | N/A | N/A | N/A | 16 y | 9 m | 17 y | 2 y  8 m |
| Proband | Sparrow  et al. 2006 | Lefebvre  et al. 2018  (1) | Lefebvre  et al. 2018 (2) | Takeda  et al. 2018 | Otomo  et al. 2019 | Schuhmann et al. 2021 | Wengryn  et al. 2023 |

**Supplemental Materials**

Table S2: Clinical Data Recorded for Each SCD3 Proband. N/A, Not Available; N/A†- Radiographs present but did not assess. AS:H Ratio: Arm-Span to Height Ratio. M: Male. m: Months. SD: Standard Deviation. y: years.
