# Supplementary material for: Integrative analysis of Lunatic Fringe variants associated with spondylocostal dysostosis type‐III
Source: FASEB J. 2024 Jun 25;38(13):e23753. doi: 10.1096/fj.202302651RR (PMC13688047; doi:10.1096/fj.202302651RR)
Supplement: Supplementary file 4 [file FSB2-38-e23753-s004.docx]

**Supplemental Materials**

**Figure S2:** **Orthologous and Paralogous Analysis of LFNG Variants**. A) Original Swiss-prot (sp) codes derived from UniprotKB, with associated sequence names to facilitate analysis during alignment. B) Through orthologous comparison of 162 LFNG sequences, all residues of interest (p.T149; light blue p.L156, p.W195, p.D201, p.T281, p.R286) appear evolutionarily conserved through a minimum sequence conservation of 90%. C) Paralogous comparison of human Fringe proteins LFNG, RFNG, and MFNG indicates sequence conservation of relevant residues with a conservation between sequences of 80-100%.
